# BOL-303242-X, a novel selective glucocorticoid receptor agonist, with full anti-inflammatory properties in human ocular cells

**Published:** 2009-12-08

**Authors:** Jin-Zhong Zhang, Megan E. Cavet, Karl R. VanDerMeid, Mercedes Salvador-Silva, Francisco J. López, Keith W. Ward

**Affiliations:** Pharmaceutical R&D, Bausch & Lomb Inc., Rochester, NY

## Abstract

**Purpose:**

BOL-303242-X is a novel selective glucocorticoid receptor agonist under clinical evaluation for the treatment of inflammatory skin and eye diseases. Data from in vitro and in vivo studies suggest an improved side-effect profile of this compound compared to classical glucocorticoids. The aim of this study was to determine the anti-inflammatory effect of BOL-303242-X in ocular cells.

**Methods:**

Four primary human ocular cell cultures, including human conjunctival fibroblasts (HConFs), human corneal epithelial cells (HCEpiCs), human optic nerve astrocytes (HONAs), and human retinal endothelial cells (HRECs), as well as a human monocytic cell line, THP-1, were challenged with either lipopolysacharide (LPS) or interleukin-1ß (IL-1ß). Luminex technology was used to determine the effect of BOL-303242-X on LPS- or IL-1ß-induced cytokine release and intercellular adhesion molecule-1 (ICAM-1) levels. Effects of BOL-303242-X on nuclear factor kappa B (NFκB) and mitogen-activated protein kinase (MAPK) in HCEpiCs were also assessed by measuring inhibitory kappa B protein-α (IκB-α), phosphorylated p65 NFκB, and MAPK levels by western blotting. Dexamethasone (DEX) or triamcinolone acetonide (TA) was used as the control.

**Results:**

LPS or IL-1ß induced multiple cytokine release in all cell types studied. BOL-303242-X significantly reduced LPS- or IL-1ß-induced inflammatory cytokine release in a dose-dependent manner, including granulocyte colony-stimulating factor (G-CSF), IL-1ß, IL-6, IL-8, IL-12p40, monocyte chemotactic protein-1 (MCP-1), and tumor necrosis factor-α (TNF-α). BOL-303242-X showed activity and potency comparable to that observed for DEX or TA. A statistically significant inhibitory effect of BOL-303242-X was observed at doses ranging from 1 to 100 nM in HConFs, HCEpiCs, HONAs, and THP-1. The IC_50_ values for these effects were in the low nM range. BOL-303242-X also significantly reduced LPS-induced IL-1ß release and ICAM-1 levels in HRECs. Furthermore, BOL-303242-X inhibited IL-1ß-induced decreases in IκB-α levels, as well as IL-1ß-induced phosphorylation of NFκB, p38, and c-Jun-N-terminal kinase (JNK) MAPKs in HCEpiCs.

**Conclusions:**

BOL-303242-X acts as a potent anti-inflammatory agent in various primary human ocular cells with similar activity and potency compared to classical steroids. Results also suggest that MAPK (p38 and JNK) and NFκB signaling pathways are involved in the anti-inflammatory properties of BOL-303242-X in HCEpiCs. An improved side effect profile of this novel SEGRA compound has been reported recently. Thus, BOL-303242-X may provide a new option for the treatment of ophthalmic conditions with an inflammatory component.

## Introduction

Activation of inflammatory pathways is a common tissue response to external pathogens and tissue damage. Inflammatory processes have been implicated in a wide variety of ocular diseases, including dry eye [1], allergy [2,3], diabetic eye disease [4], and age-related macular degeneration [5]. The role of transcription factors in modulating inflammatory processes is well known; in fact, glucocorticoid receptor modulators such as dexamethasone and prednisolone are among the most widely prescribed ocular therapeutics [6]. However, the use of steroids in ophthalmology is often associated with unwanted side effects [7,8]. Therefore, identification of new agents with reduced undesirable effects continues to represent a substantial unmet medical need.

It is believed that most side effects of steroids are mediated via transactivation-dependent gene regulation, whereas their anti-inflammatory properties are mediated largely via transrepression [9,10]. Unwanted side effects of the classical steroids used in ophthalmology correspond to the elevation of intraocular pressure (IOP) and cataract formation [7,11-13]. Selective glucocorticoid receptor agonists (SEGRAs) represent a set of molecules that preferentially regulate glucocorticoid receptor-mediated gene expression via transrepression and carry out glucocorticoid-like anti-inflammatory activities. These agents may offer a reduced side-effect profile in comparison with classical steroids [9,14,15].

BOL-303242-X (also known as ZK 245186) is a novel SEGRA compound under clinical evaluation for the treatment of inflammatory skin and eye diseases [16]. This compound belongs to the pentanolamine series of topical SEGRAs [17], and is not a steroid. It is also structurally and mechanistically unique and different from the non-steroidal anti-inflammatory drugs. In irritant contact dermatitis and T cell-mediated contact allergy models, BOL-303242-X showed anti-inflammatory efficacy after topical application similar to the classical glucocorticoids. However, it exhibited a better safety profile with regard to growth inhibition and induction of skin atrophy after long-term topical application, thymocyte apoptosis, hyperglycemia, and hepatic tyrosine aminotransferase activity [16]. In ophthalmology, recent data suggest a reduced ability for BOL-303242-X to increase IOP in normotensive rabbits when compared to dexamethasone [18]. A recent study also demonstrated that BOL-303242-X behaves as a partial agonist at increasing myocilin protein levels and gene expression in monkey trabecular meshwork cells [19]. Myocilin has been proposed to have an etiological role in steroid-induced glaucoma [20,21]. These data indicate that there is the potential for BOL-303242-X to provide a better safety profile than classical glucocorticoids when treating chronic inflammation in ocular tissues. A comprehensive evaluation of the effect of this novel SEGRA compound on inflammatory mediators, including cytokines and adhesion molecules, in key ocular tissues has not been performed to date. Therefore, the objective of these studies was to determine the anti-inflammatory properties of BOL-303242-X, on the release of important inflammatory cytokines in cultured primary human conjunctival fibroblasts (HConFs), human corneal epithelial cells (HCEpiCs), human optic nerve astrocytes (HONAs), and human retinal endothelial cells (HRECs). The responsiveness of the monocytic cell line THP-1 to inflammatory challenges was also studied as a systemic benchmark. Levels of intercellular adhesion molecule-1 (ICAM-1; a vascular endothelial inflammatory marker), were also measured in HRECs. Finally, to explore the potential signaling cascades involved in the anti-inflammatory action of this novel SEGRA compound, the effects of BOL-303242-X on the inflammatory transcription factor NFκB and MAPK signaling cascade were examined in HCEpiCs. MAPKs are serine/threonine-specific protein kinases that respond to extracellular stimuli and regulate various cellular activities. JNK and p38 MAPKs have been implicated as playing key regulatory roles in the production of proinflammatory cytokines and downstream signaling events leading to inflammation and tissue destruction [22].

## Methods

### Materials

EpiLife medium and human corneal growth supplement were purchased from Cascade Biologics (Portland, OR). Fibroblast Medium and fibroblast growth supplement were purchased from ScienCell (San Diego, CA). CS-C medium and acidic fibroblast growth factor (aFGF) were purchased from Cell Systems (Kirkland, WA). RPMI-1640 medium and DMEM medium were purchased from Invitrogen (Carlsbad, CA). Fetal bovine serum was purchased from Hyclone (Logan, UT). Charcoal/Dextran-treated fetal bovine serum (CD-FBS) and Micro BCA protein assay kit were purchased from Fisher Thermo Scientific (Waltham, MA). Dexamethasone (DEX), triamcinolone acetonide (TA), dimethyl sulfoxide (DMSO), lipopolysaccharide (LPS), and proteinase inhibitor cocktail were from Sigma (St. Louis, MO). BOL-303242-X (ZK 245186; *R*-1,1,1-trifluoro-4-(5-fluoro-2,3-dihydrobenzofuran-7-yl)-4-methyl-2-{[(2-methyl-5-quinolyl)amino]methyl}pentan-2-ol) was provided by Bayer Schering Pharma (Berlin, Germany). IL-1ß was from R&D Systems (Minneapolis, MN). Alamar Blue solution was from Biosource (Camarillo, CA). Human multiplex-cytokine and sICAM-1 Luminex kits were obtained from Millipore (Billerica, MA). Antibodies for the western blotting were purchased from Cell Signaling Technology (Danvers, MA), Santa Cruz Biotech (Santa Cruz, CA), Zymed (San Francisco, CA), and Millipore. All other reagents were purchased from standard commercial sources and were of the highest available quality and purity.

### Cells and cell treatments

Primary HCEpiCs were purchased from Cascade Biologics and maintained in EpiLife medium containing human corneal growth supplement (bovine pituitary extract 0.2% [v/v], bovine insulin [5 µg/ml], hydrocortisone [0.18 µg/ml], bovine transferrin [5 µg/ml], and mouse epidermal growth factor [1 ng/ml]). Primary HConFs were purchased from ScienCell and maintained in fibroblast medium (FM) containing fibroblast growth supplement (undisclosed formulation) and 2% FBS. Primary HRECs were purchased from Cell Systems and maintained in CS-C medium containing acidic fibroblast growth factor (aFGF) and 10% FBS. THP-1 cells were purchased from ATCC (Manassas, VA) and maintained in RPMI-1640 medium containing 10% FBS. HONAs were isolated according to Yang & Hernandez [23], characterized by immunocytochemistry using anti-glial fibrillary acidic protein (GFAP) antibody with 90–95% positive cells, and maintained in astrocyte defined serum-free medium (ADSF) containing 3% CD-FBS and G5 astrocyte supplement (insulin [5 ng/ml], human transferrin [50 ng/ml], selenite [5.2 pg/ml], biotin [10 pg/ml], hydrocortisone [3.6 pg/ml], FGF [5 pg/ml], and EGF [10 pg/ml]). These cells were all cultured in the above media, containing 100 U/ml of penicillin and 100 µg/ml of streptomycin, at 37 °C in a humidified incubator with 5% CO_2_. Prior to treatments, cells were seeded in 24-well plates and cultured until confluence. Cells were pretreated with test agents (BOL-303242-X, DEX, or TA) for 2 h, and then further treated with either vehicle (0.1% DMSO), LPS, IL-1ß, BOL-303242-X, DEX, TA, or combinations of them in basal medium (HCEpiCs and HONAs) or in basal medium plus CD-FBS (HConF, HREC, and THP-1) for 18 h. Each treatment was performed in triplicate. After conditioned media were collected for the cytokine assay by Luminex, cells were used for the Alamar Blue assay. For the ICAM-1 and IL-1ß assays of HRECs, cells were lysed in NP-40 lysis buffer (PBS containing 0.1% NP-40 and 10 µl/ml proteinase inhibitor cocktail), transferred to Eppendorf tubes, and sonicated. After centrifugation at 10,000× g for 5 min, the supernatant from each triplicate sample was transferred to a fresh Eppendorf tube for IL-1ß and ICAM-1 Luminex and protein measurements.

### Multiplex Luminex technology

Cytokine content in the culture medium or cell lysate was analyzed using multiplex Luminex technology [24-26]. Up to 26 cytokines (Eotaxin; Fractalkine; G-CSF; granulocyte macrophage colony-stimulating factor [GM-CSF]; IL-1α; IL-1β; IL-2; IL-3; IL-4; IL-5; IL-6; IL-7; IL-8; IL-10; IL-12(p40); IL-12(p70); IL-13; IL-15; IFN-γ; interferon-inducible protein-10 [IP-10]; MCP-1; macrophage inflammatory protein-1β [MIP-1α]; transforming growth factor-α [TGF-α]; TNF-α, regulated upon activation, normal T-cell expressed, and secreted [RANTES]; and vascular endothelial growth factor [VEGF]) were measured according to the manufacturer's instructions. Data were analyzed using a Luminex 200 (Luminex, Austin, TX) and Beadview software v1.0 (Upstate Cell Signaling Solutions, Temecula, CA). Standard curves for known concentrations of recombinant human cytokines were used to convert median fluorescence intensities (MFI) to cytokine concentrations in pg/ml. Only the linear portions of the standard curves were used to quantify cytokine concentrations, and in instances where the fluorescence reading exceeded the linear range of the standard curve, an appropriate dilution was performed to ensure that the concentration was in the linear portion of the curve. IL-1ß and ICAM-1 concentrations in the cell lysate were normalized based on the protein content of the sample and expressed as pg/mg protein.

### Western blot analysis for IκB-α, NFκB, and MAPKs

Cells were pretreated with vehicle, BOL-303242-X or DEX for 2 h, and then further treated with vehicle, IL-1ß, or either IL-1ß plus BOL-303242-X or DEX in basal EpiLife medium for 10 min or 30 min. Cells were washed with ice-cold PBS and lysed in cell lysis buffer (62.5 mM Tris-HCl, pH 6.8, 2% sodium dodecyl sulfate [SDS], 10% glycerol, and 1:1,000 proteinase inhibitor cocktail). Cells were sonicated, and centrifuged at 12,000 rpm. Protein concentration was determined using the Micro BCA protein assay kit. Proteins in aliquots of cell lysate (approximately 30 µg protein) were separated by SDS-polyacrylamide electrophoresis (SDS-PAGE) on 10% gels and transferred to nitrocellulose membranes. Membranes were blocked with 1% BSA and exposed to rabbit anti-phospho-p38 (Thr180/Tyr182) antibody (Cell Signaling), rabbit anti-phospho-p65 NFκB (Ser536) antibody (Cell Signaling), rabbit anti-IκB-α antibody (Santa Cruz Biotech), or rabbit anti-phospho-JNK (Thr183/Tyr185) antibody (Santa Cruz Biotech). The blots were washed, and exposed to horseradish peroxidase-conjugated anti-rabbit secondary antibody. After washing, blots were incubated in enhanced chemiluminescence solutions and chemiluminescent bands were visualized using the FluorChem imaging system (Alpha Innotech, San Leandro, CA) [25]. Blots were then stripped and re-probed as appropriate with rabbit anti-p38 antibody (Cell Signaling), rabbit anti-JNK antibody (Santa Cruz), rabbit anti-p65 NFκB antibody (Cell Signaling), or mouse anti-keratin K3/K76 antibody (Millipore; a specific marker of corneal epithelial cells), as loading controls. The experiment was repeated three to four times. Analysis of western blot band density for phosphorylated and non-phosphorylated p65 NFκB, JNKs, and p38, as well as keratin K3/K76 in captured digital images, was done using the Alpha-Innotech Chemi-Imager software **(**Alpha Innotech**)**. Levels of phosphorylated p65 NF-κB, JNKs, and p38 were normalized to non-phosphorylated p65 NF-κB, JNKs, and p38 from the same sample on the same membrane. Levels of IκB-α protein were normalized to keratin K3/K76. For quantitation of JNK phosphorylation, the densities of two JNK isoforms, JNK1 (46 kDa) and JNK2/3 (54 kDa), were determined separately and then summed together. The experiment was repeated three to four times.

### Cellular metabolic activity

Cellular metabolic activity was determined by the Alamar Blue assay [27]. Briefly, after removal of medium, cells were incubated with 1:10 diluted Alamar Blue solution for 3 h at 37 °C in a humidified incubator with 5% CO_2_. The plate was read fluorometrically by excitation at 530–560 nm and emission at 590 nm. Relative fluorescence units (RFU) were used to determine cell metabolic activity.

### Data analysis and statistics

Data were expressed as means±SEM unless otherwise indicated. Statistical analysis was conducted after evaluating the data for compliance with normality and variance homogeneity criteria. If these criteria were not met, data were Box-Cox-transformed and then the analysis was completed on the transformed data. Comparison of treatment effects across groups was performed using two-way analysis of variance (ANOVA) followed by the Tukey-Kramer test (JMP 7 software, SAS Institute, Cary, NC) on either raw or Box-Cox-transformed data [28,29] using either LPS or IL-1ß treatment as a reference. For all assays, a p≤0.05 was predetermined as the criterion of statistical significance. A Student’s t-test on either raw or Box-Cox-transformed data [28,29] was used to determine whether LPS or IL-1ß was effective at increasing cytokine release compared to the vehicle control. Dose-response curve data were fitted to a re-parameterized four-parameter logic equation [30], and this equation, below, permitted estimation of the IC_50_ values for each drug treatment.

$$y\;=\text{ Min }+\;\left(\text{Max }–\text{ Min}\right)/(\text{1}+\;e^{\text{Slope }.\;}{}^{\left[\text{logEC5}0\;–\text{ log1}0\left(\text{X}\right)\right]})$$

Where Max is maximal response, Min is minimal response, *e* is base for natural logs, slope is the Hill Coefficient • natural log of 0.1, log EC_50_ is the base 10 log of the EC_50_ and log x is the base 10 log of dose.

Statistical analysis of western blot data was performed using one-way ANOVA followed by the Dunnett’s test on either raw or Box-Cox-transformed data, and a p≤0.05 was considered statistically significant. For all the experiments, the specific transformations used are described in the figure legends.

## Results

### BOL-303242-X demonstrates activity similar to classical glucocorticoids in inhibiting LPS- or IL-1ß-induced cytokine release in four different primary human ocular cells

HConFs, HCEpiCs, HONAs, and HRECs were pretreated with the test agents, BOL-303242-X, dexamethasone (DEX), or triamcinolone acetonide (TA), for 2 h, and then further treated with vehicle, LPS, IL-1ß, test agents, LPS plus test agents, or IL-1ß plus test agents for 18 h. No statistically significant effects of BOL-303242-X or dexamethasone on cellular metabolic activity, an index of cell viability, were observed in the four cell types studied. Under basal conditions (without LPS or IL-1ß stimulation), cytokine levels in the conditioned medium were very low, with almost all the cytokines studied below the limits of detection by Luminex technology. Furthermore, no statistically significant effect on cytokine release from either BOL-303242-X or the two glucocorticoids was observed.

**Figure 1:**
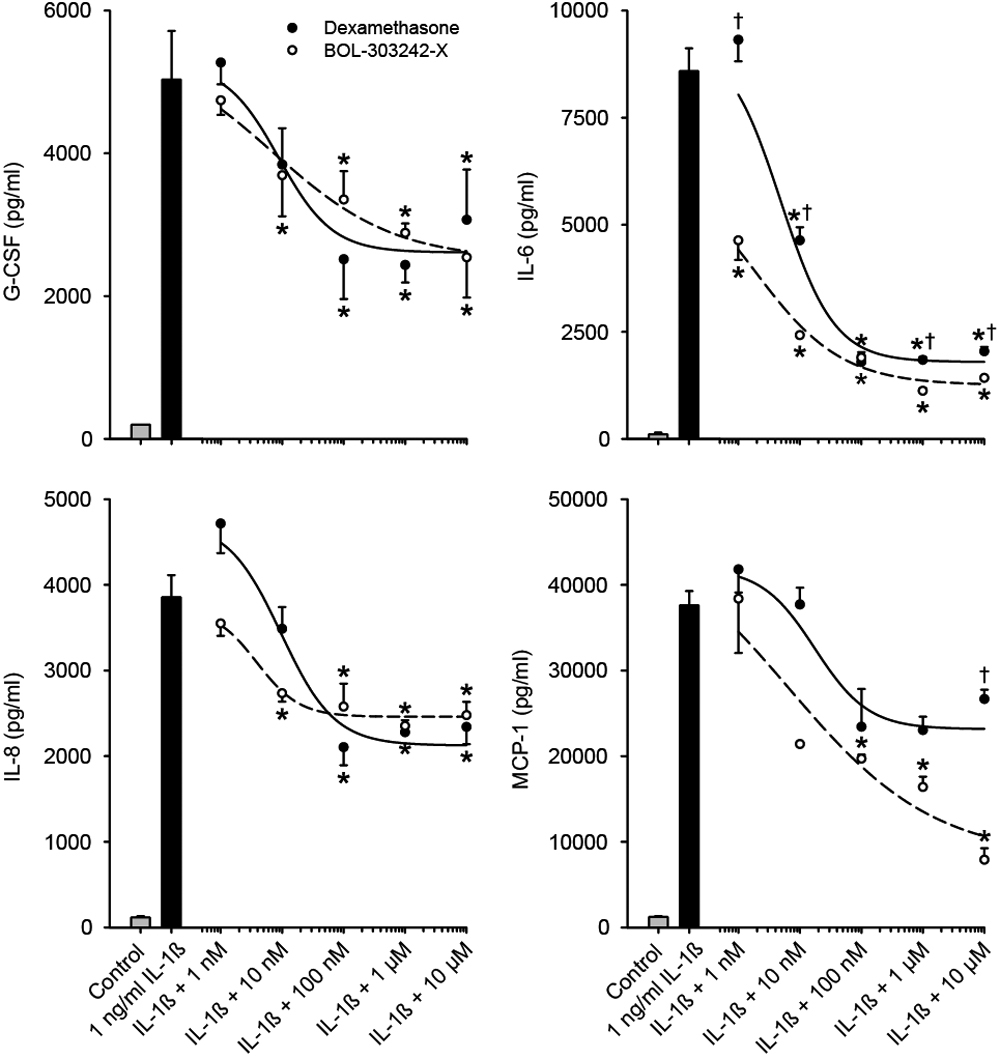
BOL-303242-X demonstrates similar activity as dexamethasone (DEX) in reducing IL-1ß-induced cytokine release from human conjunctival fibroblasts. Cells were pretreated with BOL-303242-X or DEX for 2 h, and then further treated for 18 h with vehicle (0.1% DMSO), IL-1ß, BOL-303242-X, DEX, or combinations of them. Cytokine content in the conditioned media was determined by Luminex. Data are means±SEM or geometric means±SE estimated by the Taylor Series expansion for the MCP-1 data; n=3. The asterisk indicates a p≤0.05 versus that for IL-1ß. The dagger indicates a p≤0.05 versus BOL-303242-X at same concentration. Statistical analysis was performed using two-way ANOVA followed by the Tukey-Kramer test on raw data for G-CSF and IL-8. Data were elevated to the power of 0.4 for IL-6 and by taking the logarithm of MCP-1 data. A Student’s t-test was used to determine whether IL-1ß was effective at increasing cytokine release. The solid bar is statistically significant versus the gray bar at increasing cytokine release.

BOL-303242-X significantly inhibited G-CSF, IL-6, IL-8, and MCP-1 release from HConFs at doses of 1–100 nM in a dose-dependent manner (Figure 1). The effects of BOL-303242-X were similar to those of DEX, with estimated IC_50_ values of 1 nM for DEX versus 7 nM for BOL-303242-X with G-CSF, 5 nM for DEX versus 2 nM for BOL-303242-X with IL-6, 10 nM for DEX versus 4 nM for BOL-303242-X with IL-8, and 18 nM for DEX versus 7 nM for BOL-303242-X with MCP-1. The differences observed in IC_50_ between BOL-303242-X and DEX were not considered statistically significant because confidence limits on these values overlapped for all cytokines tested. Furthermore, a significantly higher activity of BOL-303242-X was observed when compared to DEX at the 1 nM, 10 nM, 1 µM, and 10 µM doses with IL-6 (Figure 1). In addition, there was a greater inhibition of MCP-1 release by BOL-303242-X as compared to DEX at 10 µM (Figure 1). Together, these data demonstrate the full anti-inflammatory properties of BOL-303242-X in HConFs.

**Figure 2:**
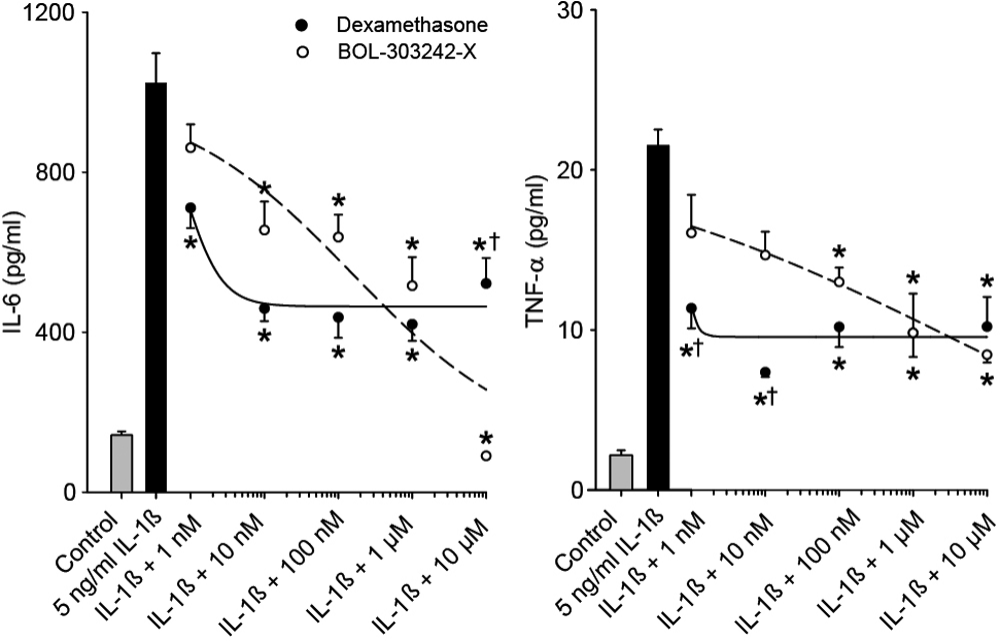
BOL-303242-X demonstrates similar activity as dexamethasone (DEX) in reducing IL-1ß-induced cytokine release from human corneal epithelial cells. Cells were pretreated with BOL-303242-X or DEX for 2 h, and then further treated with vehicle (0.1% DMSO), IL-1ß, BOL-303242-X, DEX, or combinations of them for 18 h. Cytokine content in the conditioned media was determined by Luminex. Data are means±SEM; n=3. The asterisk indicates a p≤0.05 versus IL-1ß, and the dagger indicates a p≤0.05 versus BOL-303242-X at the same concentration. Statistical analysis was performed using two-way ANOVA followed by the Tukey-Kramer test on raw data. A Student’s t-test was used to determine whether IL-1ß was effective. The solid bar is statistically significant versus the gray bar at increasing cytokine release.

In HCEpiCs, BOL-303242-X significantly inhibited IL-1β-induced IL-6 release at 10 nM and TNF-α release at 100 nM, in a dose-dependent manner (Figure 2). Whereas at lower doses, DEX induced significantly greater effects than BOL-303242-X for TNF-α, both compounds showed similar activity at reducing IL-6 and TNF-α levels at the highest doses tested (Figure 2), indicating the full agonist activity of the compound as an anti-inflammatory agent.

**Figure 3:**
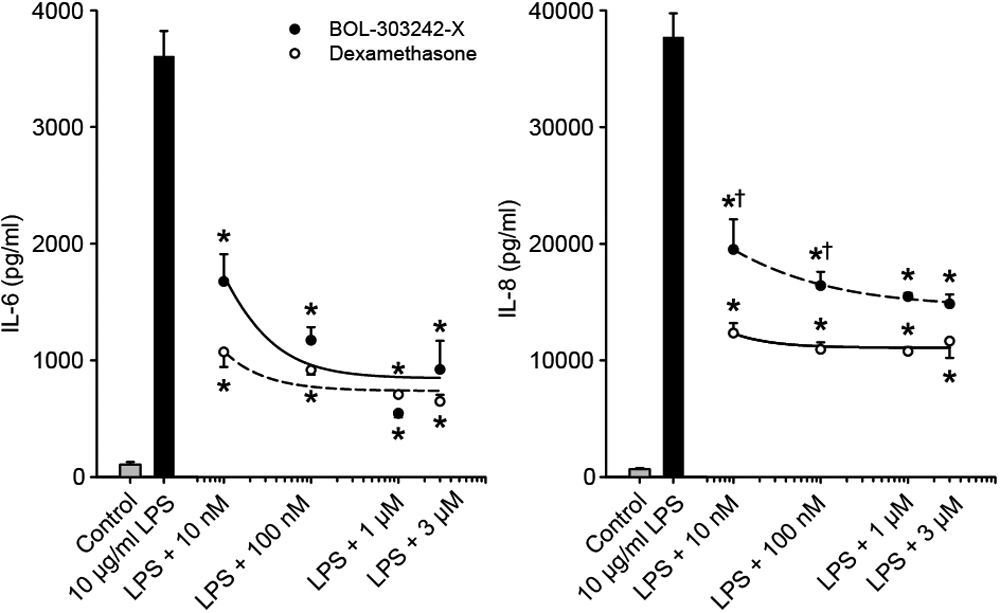
BOL-303242-X demonstrates similar activity as triamcinolone acetonide (TA) in reducing LPS-induced cytokine release in human optic nerve astrocytes. Cells were pretreated with BOL-303242-X or TA for 2 h, and then further treated with vehicle (0.1% DMSO), LPS, BOL-303242-X, TA, or combinations of them at the indicated doses for 18 h. Cytokine content in the conditioned media was determined by Luminex. Data are means±SEM; n=3. The asterisk indicates a p≤0.05 versus LPS. The dagger indicates a p≤0.05 versus TA at the same concentration. Statistical analysis was performed using two-way ANOVA followed by the Tukey-Kramer test on raw data. A Student’s t-test was used to determine whether LPS was effective at increasing cytokine release. The solid bar is statistically significant versus the gray bar.

In HONAs, both BOL-303242-X and TA inhibited LPS-induced IL-6 and IL-8 release, and a statistically significant inhibitory effect on IL-6 and IL-8 was observed at doses as low as 10 nM (Figure 3). At this dose, the activity of both compounds was very close to maximal, making it difficult to estimate IC_50_ values (<10 nM). In HONAs, BOL-303242-X behaved as a full anti-inflammatory agent, with efficacies comparable to those observed for TA in these cytokines (Figure 3), as seen in HConFs and HCEpiCs.

**Figure 4:**
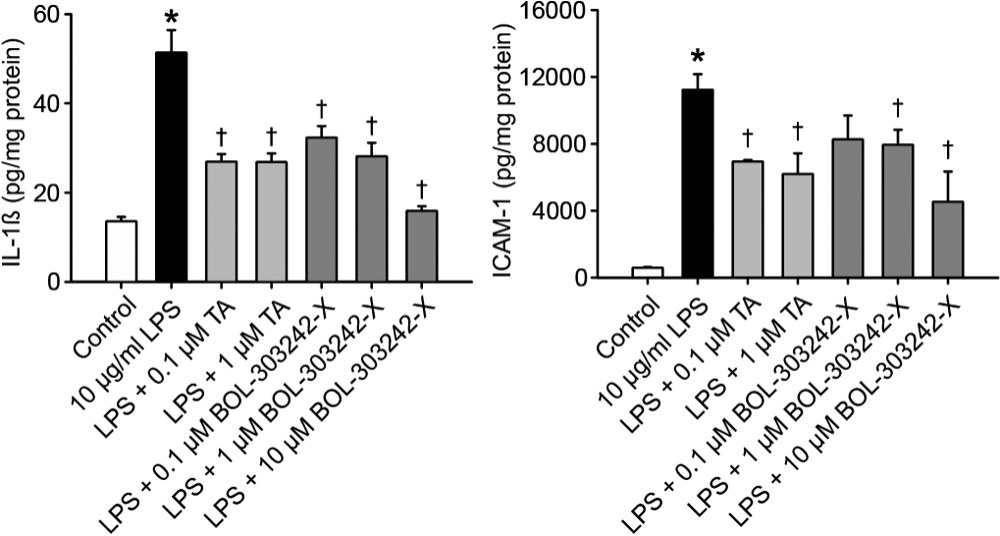
BOL-303242-X demonstrates similar activity as triamcinolone acetonide (TA) in reducing LPS-induced IL-1ß and ICAM-1 levels in human retinal endothelial cell lysates. Cells were pretreated with BOL-303242-X or TA for 2 h, and then further treated with vehicle (0.1% DMSO), LPS, BOL-303242-X, TA, or combinations of them at the indicated doses for 18 h. Cytokine and ICAM-1 content in the cell lysates was determined by Luminex, and protein content in the lysates by the Micro BCA assay. Data are means±SEM; n=3. The asterisk indicates a p≤0.05 versus the control, and the dagger indicates a p≤0.05 versus LPS. Statistical analysis was performed using one-way ANOVA followed by the Tukey-Kramer test on raw data.

BOL-303242-X and TA both inhibited LPS-induced increases in cellular levels of IL-1ß and ICAM-1 in HRECs, and a statistically significant reduction of LPS-induced increases in the cellular levels of IL-1ß and ICAM-1 was observed at doses ranging from 0.1 to 10 µM (Figure 4).

### BOL-303242-X reduces LPS-induced cytokine release in THP-1 cells

**Figure 5:**
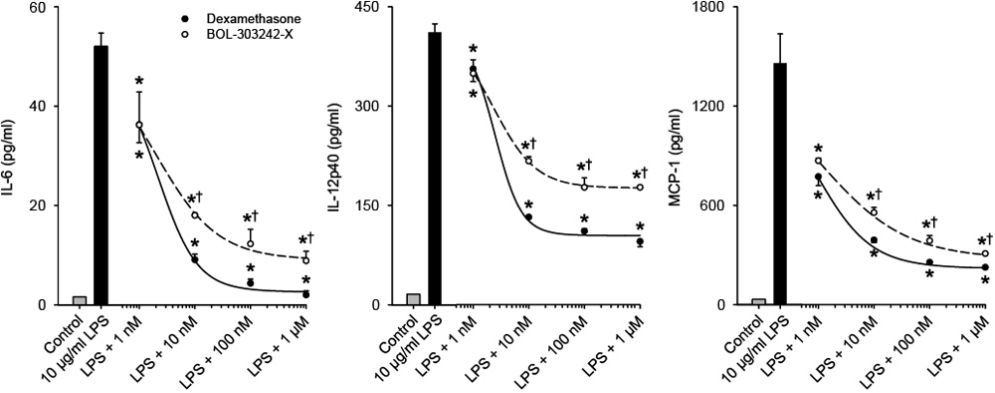
BOL-303242-X reduces LPS-induced cytokine release with similar activity as dexamethasone (DEX) from THP-1 cells. Cells were pretreated with BOL-303242-X or DEX for 2 h, and then further treated with vehicle (0.1% DMSO), LPS, BOL-303242-X, DEX, or their combinations at the indicated doses for 18 h. Cytokine content in the conditioned media was determined by Luminex. Data are means±SEM or geometric means±SE estimated by the Taylor series expansion for the MCP-1 data; n=3. The asterisk indicates a p≤0.05 versus LPS. The dagger indicates a p≤0.05 versus DEX at the same concentration. Statistical analysis was performed using two-way ANOVA followed by the Tukey-Kramer test, and data elevated to the power of 0.4 for IL-6, 0.8 for IL-12p40, and by taking the logarithm of MCP-1 data. A Student’s t-test was used to determine whether LPS was effective at increasing cytokine release. The solid bar is statistically significant versus the gray bar.

Monocytes play a critical role in the inflammatory process; therefore, the effect of BOL-303242-X on cytokine release in human THP-1 monocytes was also evaluated. Both BOL-303242-X and DEX inhibited LPS-induced IL-6, IL-12p40, and MCP-1 release in a dose-dependent manner, and a statistically significant inhibitory effect on IL-6, IL-12p40, and MCP-1 release was observed at 1 nM (Figure 5). DEX was slightly more efficacious than BOL-303242-X (86% compared to DEX for IL-6, 75% compared to DEX for IL-12p40, and 93% compared to DEX for MCP-1). However, the estimated IC_50_s for both drugs with these cytokines were comparable (2 nM for DEX versus 2 nM for BOL-303242-X with IL-6, 2 nM for DEX versus 2 nM for BOL-303242-X with IL-12p40, and 1 nM for DEX versus 1 nM for BOL-303242-X with MCP-1). Again, the differences observed in IC_50_ between BOL-303242-X and DEX were not considered statistically significant because confidence limits on these values overlapped for all cytokines tested.

### BOL-303242-X reduces MAPK phosphorylation/activation in HCEpiCs

**Figure 6:**
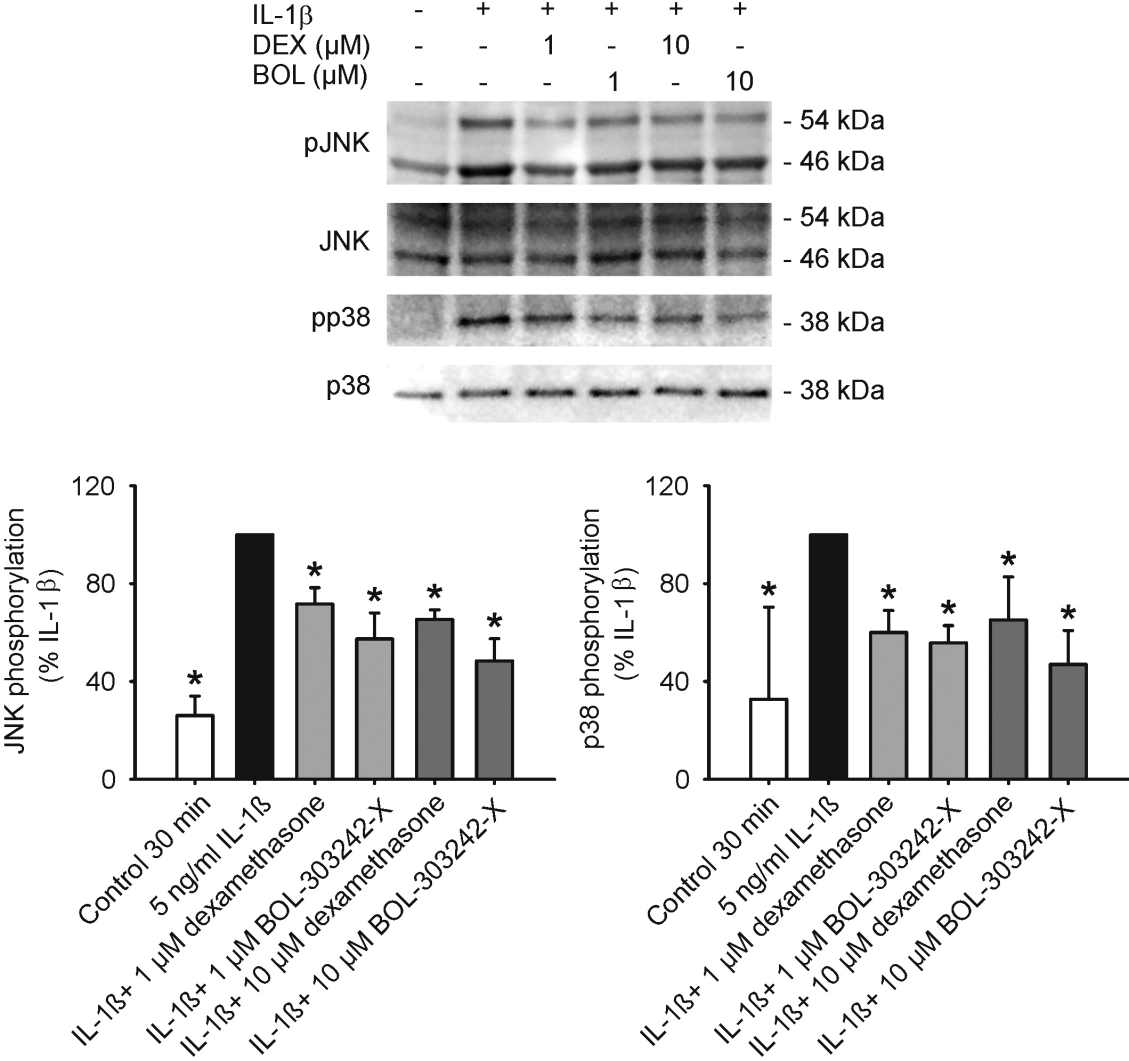
BOL-303242-X and dexamethasone (DEX) reduce IL-1ß-induced JNK and p38 MAPK phosphorylation in human corneal epithelial cells. Cells were pretreated with vehicle, BOL-303242-X, or DEX for 2 h, and then further treated with vehicle, IL-1ß, IL-1ß plus BOL303242-X, or DEX in basic EpiLife medium for 30 min. Cell lysates were prepared, resolved by SDS-PAGE, and western blotting was performed with phospho and total p38 or JNK antibodies. The top images show representative western blots for phosphorylated JNK (pJNK), total JNK, phosphorylated p38 (pp38), and total p38. At the bottom, the results of the quantification by densitometry and phosphorylated:total JNK or p38 ratios are shown for the pJNK (left panel) and pp38 (right panel) blots. The data are expressed as % of IL-β. Data are means±SEM from three to four independent experiments. The asterisk indicates a p≤0.05 versus IL-1ß. One-way ANOVA followed by the Dunnett’s test on data elevated to the power of 0.8 for pJNK and on raw data for p38. In the figure, BOL represents BOL-303242-X.

To investigate the potential mechanisms underlying the anti-inflammatory activity of BOL-303242-X, its effects on the MAPKs p38 and JNK were determined in HCEpiCs. Immunoblotting was performed using phospho-specific antibodies against JNKs at Thr183/Tyr185, and against p38 at Thr180/Tyr182. These sites have been demonstrated to be associated with the activation state of these proteins [31]. Treatment of HCEpiCs with IL-1β for 30 min significantly increased the phosphorylation state of JNK and p38 MAP kinases (Figure 6). IL-β-induced phosphorylation of p38 and JNKs was significantly reduced by BOL-303242-X or DEX at both 1 µM and 10 µM doses (Figure 6). The activity of both compounds was comparable (107% compared to DEX at 1 µM [100%] for p38, 128% compared to DEX at 10 µM [100%] for p38, 121% compared to DEX at 1 µM [100%] for JNKs, and 126% compared to DEX at 10 µM [100%] for JNKs).

### BOL-303242-X reduces NFκB phosphorylation and increases levels of IkB in HCEpiCs

**Figure 7:**
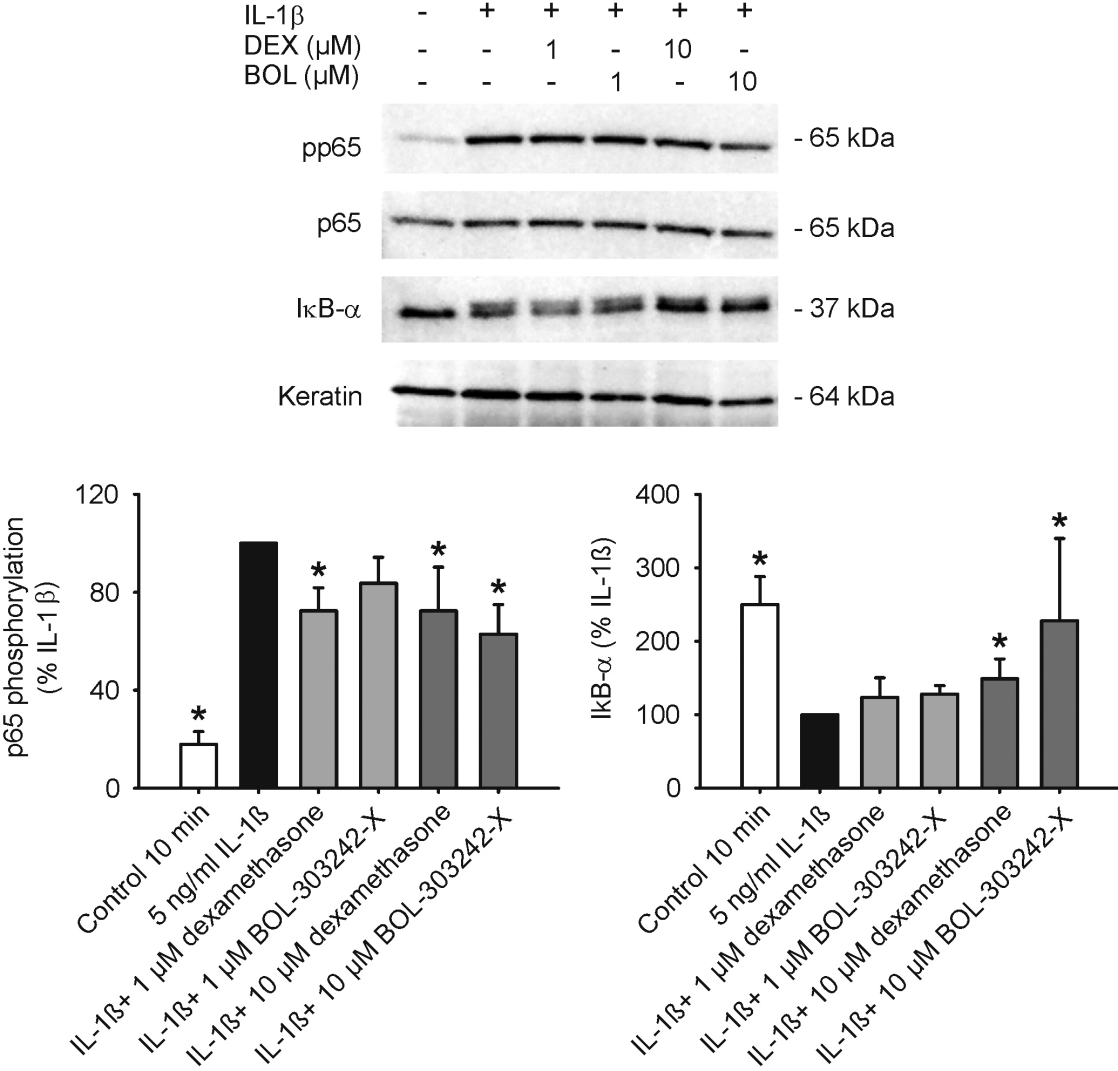
Effects of BOL-303242-X and dexamethasone (DEX) on p65 NFκB phosphorylation and IκB-α protein levels in human corneal epithelial cells. Cells were pretreated with vehicle, BOL-303242-X, or DEX for 2 h, and then further treated with vehicle, IL1ß, IL-1ß plus BOL-303242-X, or DEX in basic EpiLife medium for 10 min. Cell lysates were prepared and resolved by SDS-PAGE. Western blotting was performed with phospho-p65 NFκB followed by p65 NFκB antibodies, or with IκB-α followed by keratin antibodies. The top images show representative blots for phosphorylated p65 NFκB (pp65 NFκB), total p65 NFκB, IκB-α, and keratin. At the bottom, the results of the densitometric quantification of the blots for pp65NFκB (left panel) and IκB-α (right panel) phosphorylated:total p65 NFκB or IκB-α:keratin ratios are shown. The data are expressed as % of IL-β. Data are means±SEM from three to four independent experiments. The asterisk indicates a p≤0.05 versus IL-1ß. One-way ANOVA followed by the Dunnett’s test on raw data for pNFκB and IκB-α. In the figure, BOL represents BOL-303242-X.

The effect of BOL-303242-X on the NFκB signal transduction cascade was investigated by measuring NFκB phosphorylation and IκB-α degradation. Treatment of HCEpiCs with IL-1β for 10 min significantly increased the phosphorylation state of p65 NFκB at Ser536, a site that has been demonstrated to be associated with NFκB’s activation state [32,33]. Under basal conditions, NFκB is prevented from entering the nucleus by its interaction with IκB; therefore, a key step in the activation of NFκB is the degradation of IkB [33]. After IL-1β treatment, there was a significant decrease in cellular IκB-α levels, consistent with IκB-α degradation. IL-β-induced p65 NFκB phosphorylation at Ser536 was significantly reduced by 10 µM BOL-303242-X and both 1 µM and 10 µM DEX (Figure 7). Moreover, 10 µM BOL-303242-X and DEX significantly increased the levels of IκB-α in the cell lysate (Figure 7). These effects would be expected to result in an effective reduction of the ability of NFkB to induce inflammation by combining these two mechanisms; i.e., there would be reduction of transcription factor phosphorylation and elevation of levels of the inhibitor IκB.

## Discussion

Inflammation is a local, generally non-specific, defensive response to tissue damage caused by physical, chemical, immunological, or microbial stimuli [34]. Whereas inflammation is a physiological response, and in general is beneficial, excessive or chronic inflammatory processes result in tissue destruction and disease states. Among the various therapeutic agents that have been used to modulate inflammatory processes [35], glucocorticoids have been successfully used for the past 50 years [7]. The disorders commonly receiving glucocorticoid treatment include acute and chronic ophthalmic conditions with an inflammatory component such as dry eye [1], allergy [2,3], conjunctivitis [36], anterior uveitis [37], postoperative infection and inflammation [38], and diabetic eye disease [4,39-43]. However, the use of these synthetic and potent glucocorticoids in ophthalmology is limited by undesirable side effects, such as cataract formation and intraocular pressure elevations [11,12]. These ocular side effects are associated particularly with the long-term use of these agents [11,12].

SEGRAs represent a new class of glucocorticoid receptor modulators. These agents cause the glucocorticoid receptor to adopt a conformation such that the transcription complex preferentially supports transrepression-mediated anti-inflammatory effects over the transactivation-mediated effects [9,17,44,45]. Since most of the steroid-associated side effects are thought to be due to transactivation-mediated activities of the glucocorticoid receptor, SEGRA compounds are expected to be safer than classical glucocorticoids. A recent study has shown that ZK 245186 (BOL-303242-X) is a potent anti-inflammatory agent for the topical treatment of inflammatory skin diseases, and it has demonstrated fewer side effects than classical glucocorticoids [16]. Data from our group also suggest that BOL-303242-X offers an improved ocular side effect profile. Myocilin is a protein normally detected in the trabecular meshwork [46,47]. It has been reported that glucocorticoids increase myocilin expression in trabecular meshwork cells, and that this protein may have an etiological role in steroid-induced glaucoma [20,21]. We have found that BOL-303242-X behaves as a partial agonist compared both to dexamethasone at increasing myocilin protein levels and to the gene expression of cultured monkey trabecular meshwork cells [19,48]. These data suggest that BOL-303242-X may offer a better side-effect profile than glucocorticoids when looking at IOP elevations. This has been evaluated in in vivo animal studies, which indicated a reduced ability for the compound to increase IOP in normotensive rabbits when compared to dexamethasone [18].

Although the glucocorticoid action in inflammatory diseases is not fully understood, it is well documented that glucocorticoids inhibit the transcription of the majority of inflammatory cytokines and chemokines involved in the inflammatory process, including eotaxin, GM-CSF, IL-1ß, IL-2, IL-3, IL-4, IL-5, IL-6, IL-8, IL-11, MCP-1, MIP-1, MIP-3, MIP-4, MIP-1α, RANTES, and TNF-α, in a variety of cell types [10]. Glucocorticoids also have been shown to have a direct inhibitory effect on the expression of adhesion molecules such as ICAM-1 in endothelial cells and other cell types [10]. Elevated levels of inflammatory cytokines on the ocular surface have been reported in patients with dry eye and allergies [1,2]. Increased levels of IL-1ß and TNF-α have been detected in the serum and vitreous of patients with diabetic retinopathy [43,49,50], and these cytokines have been shown to induce inflammatory responses in HRECs [50]. In the current study, BOL-303242-X inhibited the release of multiple cytokines after either an LPS or IL-1ß challenge in four different ocular cell types (HConF, HCEpiC, HREC, and HONA), including some key proinflammatory cytokines such as IL-1ß, IL-6, IL-8, MCP-1, and TNF-α. More importantly, the observed anti-inflammatory activity in these ocular cells was comparable to that observed for two classical glucocorticoids, dexamethasone and triamcinolone acetonide, in terms of efficacy and potency, when tested under the same conditions. Furthermore, the anti-inflammatory effects observed in the cultured ocular cells were comparable to those observed in THP-1, a human monocytic cell line that has been widely used to study cytokine expression and release.

The promoter or enhancer regions of most inflammatory genes do not contain the classical GRE that are part of glucocorticoid responsive genes [51]. Therefore, it is believed that the transrepression-mediated anti-inflammatory effect of glucocorticoids does not involve direct interaction between glucocorticoid receptors and the promoter regions of proinflammatory genes. Instead, liganded and dimerized glucocorticoid receptors interact with transcription factors such as NFκB and activator protein-1 (AP-1), as well as several other cellular signaling molecules, impairing or reducing their ability to increase transcription of inflammatory genes [10,52,54]. Among these signaling pathways, mitogen-activated protein kinase (MAPK) cascades play a key role in the regulation of inflammatory gene expression [7,10,51]. In mammalian cells, three distinguishable MAPK cascades have been identified so far, consisting of the extracellular signal-regulated kinase 1 and 2 (ERK1/2), which preferentially regulates cell growth and differentiation, as well as JNK and p38 MAPK, which function mainly in stress responses like inflammation and apoptosis [22]. Glucocorticoids may inhibit AP-1 and NFκB activities via JNKs, which are known to activate these transcription factors [55,56]. Multiple inflammatory genes, including GM-CSF, IL-1ß, IL-6, IL-8, and TNF-α, are regulated by p38 MAKP, which stabilizes their mRNA [57]. There is increasing evidence that glucocorticoids may interfere with the MAPK cascades via mitogen-activated protein kinase phosphatase 1 (MKP-1), by increasing the expression of the MKP-1 gene and attenuating proteasomal degradation of MKP-1 [58].

A parallel pathway to activation of MAPK in response to inflammatory signals involves a series of kinases. Activation of these kinases leads to nuclear translocation of the NFκB. There are multiple mechanisms by which glucocorticoids may inhibit this transcription factor. Glucocorticoids can prevent the binding of NFκB to its target DNA, repress the activity of the transcription factor, and/or prevent recruitment of coactivators required for its activity. NFκB activity can also be regulated indirectly through increased synthesis of its inhibitor, IκB. Our data indicate that both BOL-303242-X and dexamethasone inhibit IL-1ß-induced reduction in IκB-α levels and IL-1ß-induced increase in p65 NFκB phosphorylation.

In summary, as an anti-inflammatory agent, BOL-303242-X is potent and fully efficacious when compared with classical glucocorticoids. The compound demonstrates dose-dependent inhibitory effects on LPS- or IL-β-induced multiple inflammatory cytokine release in primary human ocular cell cultures, including conjunctival fibroblasts, corneal epithelial cells, optic nerve astrocytes, and retinal endothelial cells. Our observations, together with data indicating that the compound acts as a partial agonist to increase myocilin protein and mRNA levels when incubated with monkey trabecular meshwork cells [19], and that the compound shows a reduced propensity to elevate IOP in normotensive rabbits [18], suggest that this novel SEGRA compound may be a new option for the treatment of chronic inflammatory conditions of the eye, and may provide a beneficial side-effect profile in comparison with classical steroids.
